# Annotations

**Published:** 1893-11-25

**Authors:** 


### ANNOTATIONS.

### Curiosities of Alcoholism.

At the recent meeting of the Study of Inebriety Society Dr. T. L. Wright, of Ohio, read a paper on the "Special Influences of Alcohol," moral and otherwise. Among other things, he mentioned some curious facts connected with the habits of what may be called "alcohol thieves." It is recognised by alcohol specialists that certain persons almost invariably try to steal when drunk who show no tendency to theft at any other time. Not only so, but the alcohol thief very generally "goes for" some particular object when the thieving mania is upon him. One man, for example, when drunk showed a uniform partiality for Bibles. He stole Bibles invariably when he could get at them. Another had a precisely sim[illegible] passion for spades. A young man, who [illegible]nably had been previously unknown at [illegible]ce court, stole a horse when drunk, an[illegible] in the presence of its owner. As he [illegible] with the animal he told his name and [illegible]domina[illegible] to persons whom he met on the road. He fibeen irold the horse. When the case came on for trial ware, [illegible]prit was acquitted on the ground of drunkenness and consequent irresponsibility. Thus encouraged he got drunk again, and stole a second horse. A year's imprisonment was now tried instead of acquittal. Coming out of prison he got drunk once more, and with insane consistency took possession of a third horse which did not belong to him. On this occasion the Court once more resolved to try the effect of pardon. But pardon was once more as useless as punishment, for a fourth horse was stolen in a drunken fit in a very short time. What was the end of this young man's career? That is not yet known, for he is said at this moment to be spending a term of imprisonment in the hope that reflection may turn him into a teetotaler—"a consummation devoutly to be wished."

### Kirkheaton Sanitary Matters.

Mr. G. A. Broadbent, surveyor to the Local Board of Kirkheaton, writes to the *Huddersfield Chronicle* protesting against the article in The Hospital which summarises Dr. Barry's report to the Local Government Board on the sanitary state of the district. Some of our remarks, he says, "are entirely uncalled for, and will have a tendency to lessen the chance of the township being a business and residential district if they are not refuted." This we can quite understand, and we will gladly give all possible publicity to the refutation. At the same time, it is only fair to state that no assertion was made which was not drawn from Dr. Barry's report, and the comments made on these facts were in no degree unreasonable. The Hospital has no ill-will to Kirkheaton, and its selection as an example of the work of the Local Government Board was only, as the *Huddersfield Chronicle* headed the article it quoted from our columns, "A Local Illustration" of a condition of affairs not so uncommon as is desirable. What, then, are the accusations which Mr. Broadbent desires to refute. One is, that Kirkheaton is a poor place. We put forward the statement as an excuse for the dilatoriness of Kirkheaton in matters sanitary, but apparently our intentions have not been appreciated. But we are not primarily responsible for it. The author is Mr. E. A. Beaumont, clerk to the Kirkheaton Local Board, who, in a letter dated April 10th, 1890, which deprecates the inquiry which the Local Government Board thought needful, says as an excuse for things amiss, "the district is poor, and cannot be pushed on so quickly as a town." The clerk and the surveyor to the Local Board thus contradict each other; and we can only hope that things have so much changed in Kirkheaton during the last three and a half years that Mr. Broadbent's denial that it is a poor place is true. "With respect to trade," he says, "I am pleased to say that they are busy at Shop Lane Mills." Is this the mill alluded to by Dr. Barry, who says that 300 workpeople are employed there, and that "21 water-closets are provided, five of which discharge directly into the sewer, and sixteen into cesspools having overflow thereto?" This sewer "finally discharges into Kirk Ings Beck without any kind of treatment whatever." If this is the establishment Mr. Broadbent speaks of, we should be glad to know if its sanitary arrangements have been improved since the date of Dr. Barry's report. "With respect to the large midden," says Mr. Broadbent, "that is being remedied." We are glad to hear this, but surely in the three years which have elapsed since Dr. Barry inspected the district, even the mightiest of middens might have been demolished. Mr. Broadbent assures us that the members of the Local Board are "shrewd business men, and wide awake to the requirements of the township, and given time and opportunity, will work a wonderful change for the better government of the district." We are glad to hear this, and would point out to these gentlemen that there is no time like the present, and that in matters sanitary opportunities must be made if they do not come. For sanitation is not a matter of convenience or inconvenience merely, but of health and life itself. Mr. Broadbent considers that Kirkheaton could be made into a "beautiful residential property." Good drainage and decent arrangements for the disposal of refuse will greatly help this desirable end, and we are therefore glad to know that a sewage scheme is now under consideration.

### Death Certification and Murder.

On the 28th ult. we called attention to the momentous fact that upwards of 14,000 persons are buried in England and Wales every year whose deaths are uncertified. Well might the Select Committee of the House of Commons "feel much impressed with the serious possibilities implied in the system which permits death and burial to take place without the production of satisfactory medical evidence as to the cause of death." They were surely more than justified in declaring that "the existing procedure plays into the hands of the criminal classes." A District Registrar, however, writes to point out that in some cases deaths returned as uncertified have in reality been certified by M.D.'s of foreign Universities, possessing no English qualification, or by unqualified assistants. We have made full enquiry, and find that this statement does not materially affect the present position. Upwards of 15,100 deaths were registered in England and Wales during 1892, the causes of which were not certified either by a registered medical practitioner or by a coroner. It further appears that in a large proportion of the uncertified deaths the deceased persons had had no kind of medical attendance, although a few may have had some so-called medical aid of varying degrees of value. Our correspondent, the District Registrar, must also remember that non-registration in the case of medical men is very often the result of inability to pass qualifying examinations. Hence the safety of the community demands that Parliament shall promptly step in and put a stop to the further registration of uncertified deaths by the institution of some such system as that we formulated in our article on the 28th ult.

